# Extracapsular Dissection With Narrow-Band Imaging Using the Transnasal Endoscopic Tri-port Approach for Extracranial Trigeminal Schwannoma: A Case Report

**DOI:** 10.7759/cureus.58200

**Published:** 2024-04-13

**Authors:** Keisuke Yamamoto, Tsuyoshi Okuni, Makoto Kurose, Yukinori Akiyama, Kenichi Takano

**Affiliations:** 1 Department of Otolaryngology-Head and Neck Surgery, Sapporo Medical University School of Medicine, Sapporo, JPN; 2 Department of Neurosurgery, Sapporo Medical University School of Medicine, Sapporo, JPN

**Keywords:** narrow-band imaging, pterygopalatine fossa, extracranial trigeminal schwannoma, endoscopic transnasal surgery, extracapsular dissection

## Abstract

The pterygopalatine fossa and infratemporal fossa are often approached through an external incision because of their deep facial location, but this can present problems such as facial scarring and deformity. In schwannoma surgery, intraneural dissection is a useful surgical technique for achieving gross total resection while preserving the capsule, including the nerves. For appropriate enucleation and preservation of the functional nerve, it is indispensable to distinguish between the pseudocapsule and the tumor capsule. This case report presents a case of endonasal surgical intervention for an extracranial trigeminal schwannoma employing the tri-port approach and narrow-band imaging.

## Introduction

Trigeminal schwannomas account for 8% of skull base tumors [1], and extracranial trigeminal schwannomas account for 10% of all trigeminal schwannomas. Extracranial trigeminal schwannomas, which were previously treated by external incision, are nowadays increasingly indicated for endoscopic transpterygoid surgery [2]. The pterygopalatine fossa is a deep facial region located in the posterior wall of the maxillary sinus, where the tri-port approach provides an excellent surgical view and wide corridor without an external approach such as a gingival incision [3].

As most extracranial trigeminal schwannomas are asymptomatic and pathologically benign, the goal of treatment is to safely perform gross total resection of the tumor without neurovascular complications. Therefore, intraneural dissection is a useful surgical technique for achieving gross total resection while preserving the capsule, including the nerves [4]. To support this method, narrow-band imaging (NBI)-guided surgery is reported to be useful [5,6]. NBI is a digital-optical image-enhanced endoscopy method that enables early detection of malignancy, grade of malignancy, and diagnosis of the extent of malignancy through highlighting differences in mucosal color tone, and it is also increasingly being used for non-malignant lesions in the head and neck region [7,8]. In this report, we describe a case of an extracranial trigeminal schwannoma that was intraneurally dissected by nasal endoscopic surgery with NBI along with preservation of the infraorbital nerve.

## Case presentation

**Figure 1:**
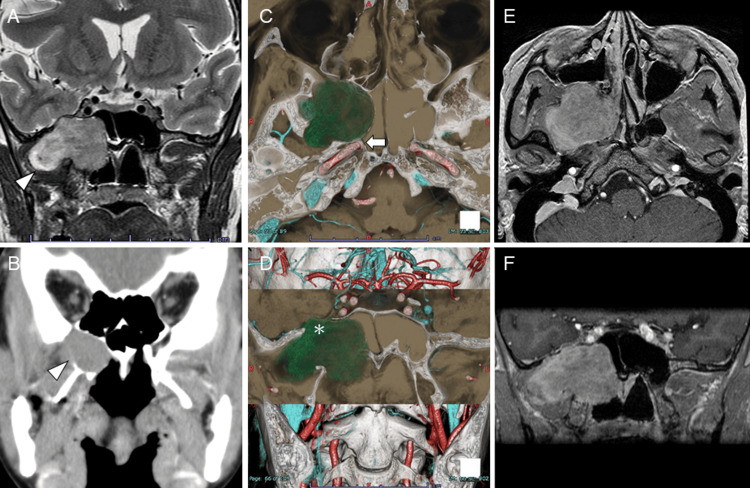
Preoperative CT and MRI images of the patient. (A) T2-weighted MRI reveals a mass lesion located on the right side of the pterygopalatine fossa. The lesion shows internal heterogeneity and moderate-to-high signal, extending into the inferior temporal fossa (arrowhead). (B) A tumor was incidentally visible in the right pterygopalatine fossa on a CT scan seven years previous, but it did not extend into the inferior temporal fossa (arrowhead). (C, D) Three-dimensional CT shows a well-defined, limbally smooth lobulated mass with a maximum size of 75 mm in the right pterygopalatine fossa to the inferior temporal fossa, with a dilated right median foramen (asterisk). The right internal carotid artery and mass are bordered by the bony wall of the carotid canal (arrow). (E, F) T1-weighted contrast-enhanced MRI shows evidence of enhancement of the mass.

A 47-year-old woman experienced sudden right-sided hearing loss. She had no history of occasional drinking or smoking. The right-sided hearing loss that was present for several days was diagnosed as recurrent acute sensorineural hearing loss and was treated with steroids by an otolaryngologist at a local hospital. An inner ear MRI scan revealed a mass lesion in the right pterygopalatine fossa (Figure 1A). The mass showed moderate-to-high signal with internal heterogeneity and diffusion limitation on T2-weighted imaging. On a retrospective review, the MRI scan showed that the mass had increased since a CT scan acquired seven years previously (Figure 1B), and the patient was referred to our hospital. The three-dimensional CT scan showed a well-defined, limbally smooth lobulated mass with a maximum size of 75 mm in the right pterygopalatine fossa to the inferior temporal fossa, dilating the right side of the foramen rotundum. The right internal carotid artery and the mass were bordered by the bony wall of the carotid canal (Figures 1C, 1D). T1-weighted, contrast-enhanced MRI showed evidence of enhancement (Figures 1E, 1F). Based on these findings, an extracranial right trigeminal schwannoma was diagnosed located in the pterygopalatine and inferior temporal fossa with no intracranial extension. Although asymptomatic, the patient requested resection because the lesion was showing a tendency for enlargement. A transnasal tissue biopsy showed bundles of spindle-shaped cells with long oval nuclei and acidophilic endoplasmic reticulum, with a partly fenestrated arrangement of the nuclei. Immunostaining revealed the spindle-shaped cells to be S-100(+) with a Ki-67 labeling rate of 1%. The diagnosis was histologically non-malignant schwannoma. At a preoperative conference with the neurosurgeon, it was decided to undertake a nasal approach with a neurosurgeon on standby without an external incision because the histopathology ruled out malignancy, the tumor did not extend intracranially, and the septal barrier with the internal carotid artery was preserved.

Surgery

**Figure 2:**
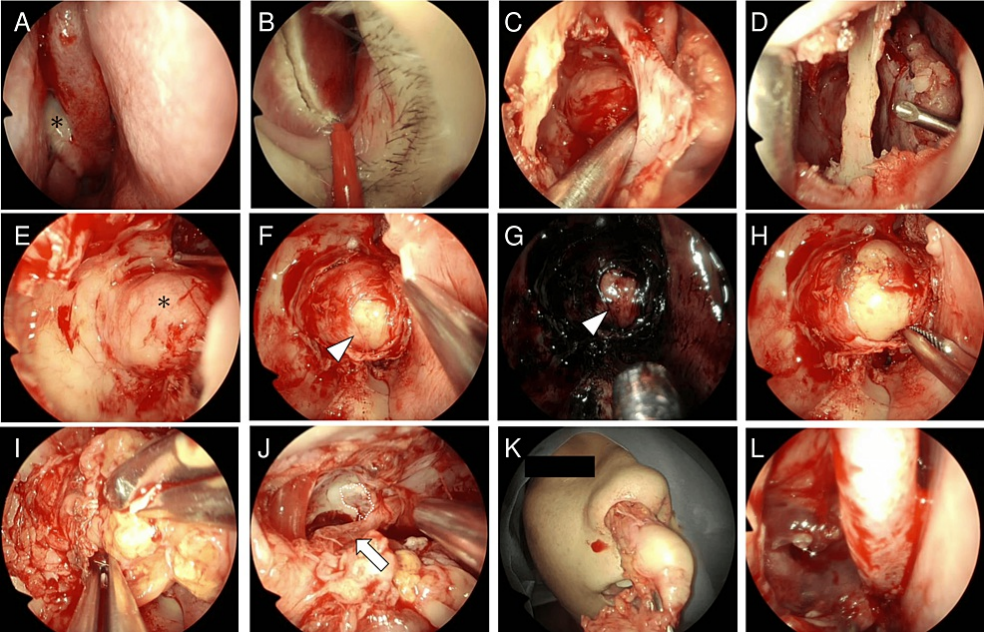
Intraoperative findings. (A) The mass was partially identified through the semilunar fissure (asterisk). (B) A left modified Killian incision was made. Following a horizontal incision along the floor of the left nasal cavity, a Killian incision and an upper horizontal incision were made in the right nasal cavity and endoscopic sinus surgery. (C) An endoscopic modified medial maxillectomy was performed to open the maxillary sinus while preserving the right piriform aperture, the right nasolacrimal duct, and the right inferior nasolacrimal duct. (D) Another port was created from the anterior wall of the maxillary sinus with preservation of the piriform aperture and infraorbital nerve. (E) Consequently, the maxillary sinus with tumor (asterisk) could be accessed through the anterior wall, right nasal cavity, and left nasal cavity across the septum. (F) Following the intraneural dissection, the pseudocapsule could be seen as red and the true tumor capsule as yellow (arrowhead) under white light illumination. (G) Under narrow-band illumination, the pseudocapsule appeared greenish-gray and the true tumor capsule (asterisk) appeared white. (H) An extracapsular dissection was performed in the layer between the pseudocapsule and true tumor capsule. (I) The assistant performed suction and countertraction from the left side. (J, K) Gross total resection was performed, preserving the maxillary artery and the supposed infraorbital nerve (arrow) following the mid-cranial defect (dotted line). (L) Fibrin glue was sprayed on the middle cranial defect.

The mass was partially identified through the semilunar hiatus (Figure 2A). A left modified Killian incision was first made (Figure 2B), followed by a horizontal incision in the mucosa along the floor of the nasal cavity for a transseptal approach. The nasal septal cartilage, perpendicular plate, and vomer were partially resected, preserving the L-strut. A Killian incision was subsequently made in the right lateral nasal septal mucosa approximately 3 mm posterior to the left side. A horizontal upper incision was made in the right lateral nasal septal mucosa to be continuous with the right Killian incision. Then, an endoscopic modified medial maxillectomy (EMMM) was performed to serve as a second port, preserving the right piriform aperture, the right nasolacrimal duct, and the right inferior nasolacrimal duct (Figure 2C). A third port was then created from the anterior wall of the maxillary sinus (while preserving the piriform aperture and infraorbital nerve), enabling access through the right external nostril to the right maxillary sinus via the anterior wall of the right maxillary sinus (Figure 2D), thereby exposing the tumor (Figure 2E). After the dissection of the pterygopalatine fossa, intraneural dissection was performed: an incision was made in the free nerve fibers and NBI was used to distinguish the true tumor capsule from the pseudocapsule. Under white light illumination, the pseudocapsule appeared red and the true tumor capsule appeared yellow, whereas under NBI, the pseudocapsule appeared greenish-gray and the true tumor capsule was white with high contrast (Figures 2F, 2G). An extracapsular dissection was performed in the layer between the pseudocapsule and the tumor capsule (Figure 2H) with the assistance of suction, and countertraction was performed through the septum (Figure 2I). Enucleation of the schwannoma was performed transnasally: a gross total resection was performed, preserving the maxillary artery and the supposed infraorbital nerve following the mid-cranial defect (Figures 2J, 2K). Fibrin glue was then sprayed on the middle cranial defect (Figure 2L). The procedure was finished by suturing the nasal septal mucosa and placing a silicone plate over the nasal septum. The operative time was 338 minutes and blood loss was minimal (Video 1).

**Video 1:** Endonasal tri-port approach with the assistance of narrow-band imaging for extracranial trigeminal schwannoma.

**Figure 3:**
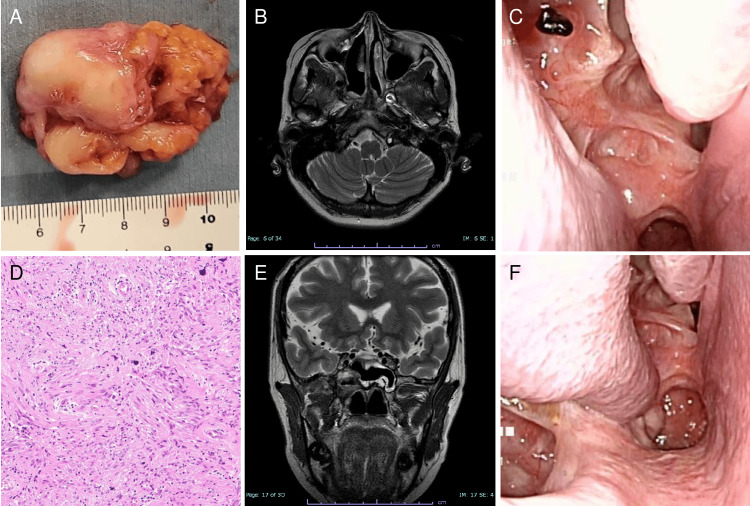
Pathological and postoperative findings. (A, D) Both the gross and microscopic findings were indicative of a schwannoma, with no malignant findings. (B, F) MRI and (C, F) fibroscopy examination 18 months postoperation showed no residual or recurrent disease.

The histopathological diagnosis was the same as the preoperative diagnosis, with no malignant findings throughout the tissue (Figures 3A, 3D). Fiberscopy and MRI performed 18 months postoperation showed no residual or recurrent disease (Figures 3B, 3C, 3E, 3F). A numbness in the right cheek area that occurred immediately after surgery gradually decreased in extent.

## Discussion

In this report, we describe a transnasal dissection of a schwannoma extending into the pterygopalatine and infratemporal fossae. This procedure was performed through multiple ports without an external incision. Importantly, the use of NBI during the intranasal dissection part of this transnasal procedure made it easier to distinguish the true capsule from the pseudocapsule, thus facilitating the preservation of the functional fascicles and preventing the sacrifice of the infraorbital nerve.

Although rather aggressive external incisional skull base surgery used to be performed for extracranial trigeminal schwannomas, transnasal endoscopic resection of extracranial trigeminal schwannomas has become available as our understanding of the complex anatomy of the skull base has developed. However, to our knowledge, there are not many publications reporting the treatment of extracranial schwannomas by transnasal endoscopy [1,9-11]. The indications for endoscopic treatment are generally the presence of a tumor involving the trigeminal ganglion (Meckel cave), V2 trunk (pterygopalatine fossa), and V3 trunk (infratemporal fossa) [2,11]. However, it is difficult to deal with major hemorrhage when performing endoscopic surgery, and careful observation of the location of the carotid artery and tumor plays a major role in the feasibility of the endoscopic approach [12]. In this case, the indications for endoscopic nasal surgery were good because the tumor was located in the pterygopalatine and infratemporal fossae without intracranial extension, and the septal barrier between the internal carotid artery and the tumor was preserved.

Approaches to the pterygopalatine and infratemporal fossa have traditionally been performed through external facial incisions, but these can cause problems with scarring and deformity. In this respect, the creation of multiple corridors to approach the pterygopalatine and subtemporal fossae using an endoscope alone was recently reported [3]. In this case, we could obtain a good approach and surgical view of the pterygopalatine fossa and the infratemporal fossa using a tri-port approach [3]. This method combined the following three approaches: transseptal access with crossing multiple incisions [13], direct approach to the anterior and lateral part of the maxillary sinus with an endoscope (DALMA) [14], and EMMM [15]. Liu et al. reported that the binostril approach provides surgical corridors for bilateral sphenoidotomy, septectomy, bilateral ethmoidectomy, bilateral frontal sinusotomy, and unilateral medial maxillectomy, and the transpterygoid approach allows access to the bilateral nasal cavity, sphenoid sinuses, ethmoid sinuses, frontal sinuses, entire cribriform plate, ipsilateral maxillary sinus, pterygopalatine fossa, posterior nasopharynx, and medial infratemporal fossa (ITF) [16]. The Caldwell-Luc Surgical Corridor enables the sublabial maxillotomy approach, allowing access to the lateral ITF. In this case, the tumor had progressed to the ITF. By adding not only EMMM but also DALMA, it was possible to obtain more extensive access to the ITF [2]. We approached the pterygopalatine and infratemporal fossae from the following three routes: the transnasal septal route, the anterior route of the nasolacrimal duct, and the anterior wall of the transmaxillary sinus. These were performed without a gingival incision, and with preservation of the middle turbinate, inferior turbinate, piriform aperture, and nasolacrimal duct. These approaches allowed us to perform four-hand surgery, which facilitated assistance from the other side, including suctioning and countertraction.

In the surgical resection of benign nerve sheath tumors, the goal is to achieve gross total resection while preserving the functional fascia and avoiding iatrogenic nerve damage. Intraneural dissection is a kind of internal neurolysis that is useful for achieving this. It includes identifying a fascicle-free site, cutting through the pseudocapsule, and dissecting through the pseudocapsule to find the correct plane so that function can be preserved. Extracapsular dissection following intraneural dissection creates a plane between the pseudocapsule and the true capsule. Subsequent enucleation involves a gross total dissection of the tumor between the pseudocapsule and the true capsule. This results in the loss of only non-functional entering/exiting fascicles and the functional fascicles are preserved [4]. NBI is frequently used when performing image-enhanced endoscopy, and Shimane et al. reported that it is useful in the series of procedures used in this case [4,6]. NBI uses two narrow-band wavelengths (blue light between 390-445 nm and green light between 530-550 nm) to visualize the microvascular structure of the mucosa according to its relative absorption of the two wavelengths by hemoglobin. In schwannomas, NBI creates a significantly higher contrast between the pseudocapsule and the tumor capsule than white light imaging and allows the pseudocapsule to be observed in depth. This makes it easier to distinguish between the pseudocapsule and the tumor capsule, leading to appropriate enucleation and preservation of the functional nerve.

There are several limitations to the procedure described in this report. Once blood adheres to the tissue, even tissue that appears white, such as tumor fascia, absorbs the light from NBI; thus, NBI must be used in an operative field with controlled hemorrhage. As NBI uses an optical device developed by Olympus, an Olympus endoscopic system should be used. Currently, other image enhancement technologies are available for endoscopy, and it is hoped that these will be investigated in the future. To our knowledge, this is the first report of the removal of an extracranial trigeminal schwannoma by a tri-port approach utilizing NBI, and it is, therefore, a preliminary report and involves only a single patient because of the rarity of these tumors. More data are needed from studies involving a larger number of patients.

## Conclusions

Surgery was performed using a tri-port approach that provided a good surgical view of the pterygopalatine fossa and the infratemporal fossa without the need for an external incision. The method combined transseptal access with crossing multiple incisions, a direct approach to the anterior and lateral parts of the maxillary sinus with an endoscope, and EMMM. We believe that an endonasal tri-port approach with the assistance of NBI is a cosmetically acceptable approach for extracranial trigeminal schwannomas that avoids facial scarring and deformity. It is also functionally acceptable because it allows preservation of the nerve of origin while facilitating gross total resection.

The use of NBI during intranasal dissection was an important part of the procedure. In the treatment of schwannomas, NBI results in high contrast between the pseudocapsule and tumor capsule, making it easier to distinguish between them, which in this case facilitated the preservation of the functional fascicles and prevented the sacrifice of the infraorbital nerve.
